# Photocatalytic Properties of Tetraphenylporphyrins Immobilized on Calcium Alginate Aerogels

**DOI:** 10.1038/s41598-017-12945-9

**Published:** 2017-10-03

**Authors:** Anna B. Solovieva, Alexander S. Kopylov, Marina A. Savko, Tatiana S. Zarkhina, Darya D. Lovskaya, Artyom E. Lebedev, Natalia V. Menshutina, Alexey V. Krivandin, Ilya V. Shershnev, Svetlana L. Kotova, Peter S. Timashev

**Affiliations:** 1 N.N.Semenov Institute of Chemical Physics, Department of Polymers and Composites, 4 Kosygin St., 119991 Moscow, Russia; 2 D.Mendeleev University of Chemical Technology of Russia, 9 Miusskaya Sq., 125047 Moscow, Russia; 3 Institute for Regenerative Medicine, I. M. Sechenov First Moscow State Medical University, 8 Trubetskaya St., Moscow, 119991 Russia; 4 Institute of Photonic Technologies, Research center “Crystallography and Photonics”, 2 Pionerskaya St., Troitsk, Moscow, 142190 Russia

## Abstract

We have prepared photocatalytic systems based on tetraphenylporphyrins (TPP) immobilized on calcium alginate solid gels in the conditions of thermal drying on air (xerogel), freeze drying in vacuum (cryogel) and supercritical drying in the supercritical carbon dioxide (scCO_2_) medium (aerogel). As a test reaction to measure the prepared systems’ efficiency, we studied tryptophan photooxidation in the aqueous medium. We have demonstrated that the systems with aerogel as a carrier exhibited the highest photocatalytic efficiency. In that case, the rate constant for the test substrate (tryptophan) oxidation exceeds the corresponding rate constants of similar systems based on xerogel and cryogel by more than 5 times. Moreover, the aerogel-based photocatalytic systems demonstrated enhanced functional stability and a possibility of multiple use of such a catalyst in tryptophan oxidation. Based on the data of small-angle X-ray scattering and thermooxidative destruction, we have made a conclusion about the relationship between the high photocatalytic activity of aerogel-immobilized TPP and formation of a developed porous aerogel structure in the conditions of drying in the scCO_2_ medium, which is stabilized due to formation of additional complex bonds of calcium ions with fragments of glycoside rings.

## Introduction

Creation of heterogeneous photocatalytic systems via immobilization of low-molecular catalysts on inorganic or polymeric carriers allows improvement of such systems’ stability and facilitation of separation of the final reaction products, as well as enhancement of the process efficiency in certain cases. The search of a proper carrier for immobilization of functionally active substances is one of the problems in the development of such systems. Inert matrices with a highly developed surface are conventionally used as carriers. One of the new types of carriers are inorganic aerogels based on metal oxides and silicon oxides^1^ which may be prepared relatively easily and are characterized by high thermal stability.

Aerogel (AEG) is a unique 3D-arranged nanostructured highly porous material with a low density (0.003–0.35 g/cm^3^), high area of the inner surface (up to 1000 m^2^/g), pore sizes of an order of several nanometers^2,3^. Some authors believe aerogels are a new state of matter, an intermediate one between the condensed state and gas state, due to the mentioned AEG bulk properties, as well as transitional (between the two states) values of density and enthalpy of the system^3–5^. AEG may have a super-low heat conductivity, super-low elastic modulus, super-low dielectric constant and the sound velocity, high specific surface area and a super-broad controlled range of the density and refraction index. Though aerogels may be prepared on the basis of organic (polymers, resins) compounds^6,7^, the most wide-spread and well-studied aerogels used in the research and technology are inorganic aerogels, primarily, based on silicon dioxide, metal oxides, carbon^8–10^. They are applied, in particular, in the development of gas and fluid filters, heat-insulating materials, heterogeneous catalysts etc. At the same time, natural polymer-based aerogels with an intrinsic biological activity allow formation of heterogeneous catalytic systems of a new type for the medicine and pharmacology, acting as *active* carriers enhancing the effect of the immobilized substances^11^. For example, one of the new directions in the creation of prolonged drug forms is introduction of biologically active substances into polysaccharide-based aerogel matrices for drug delivery systems^12–15^. Natural polysaccharides of different structures, in particular, chitosan, starch, pectin, cellulose, alginate, carragenan, agar-agar are used for such a purpose^16–19^.

The high specific surface area of polysaccharide-based aerogels combined with their bactericidal and non-toxic properties allow their application as carriers for preparation of heterogeneous photosensitizing systems. Such systems may be utilized in the photodynamic therapy of wounds, trophic ulcers, burns for repeated treatment of lesions.

In this study, we have prepared such systems by immobilization of tetraphenylporphyrin (TPP) and its derivatives, as most active photosensitizers of singlet oxygen generation, on solid calcium alginate gels. We used gels dried by different techniques (xero-, cryo- and aerogels). We have shown that the most active catalytic systems are formed by TPP immobilization on aerogels, with the activity of the prepared systems preserved during repeated use in a model process of tryptophan photooxidation in the aqueous medium.

## Results

### Photocatalytic activity of the systems “porphyrin-solid gel”

The basic result of our study on the prepared systems’ photocatalytic activity in the tryptophan oxidation consists in the finding of the highest activity of the AEG/TPP system as compared to the systems based on porphyrins immobilized on xero- and cryogels. We have shown that *k*_*eff*_ = 40.2 g/sec·mol for the AEG-TPP system, while *k*_*eff*_ = 21.0 g/sec mol and *k*_*eff*_ = 8.5 g/sec mol for the systems with TPP immobilized on cryogel and xerogel, respectively.

**Figure 1:**
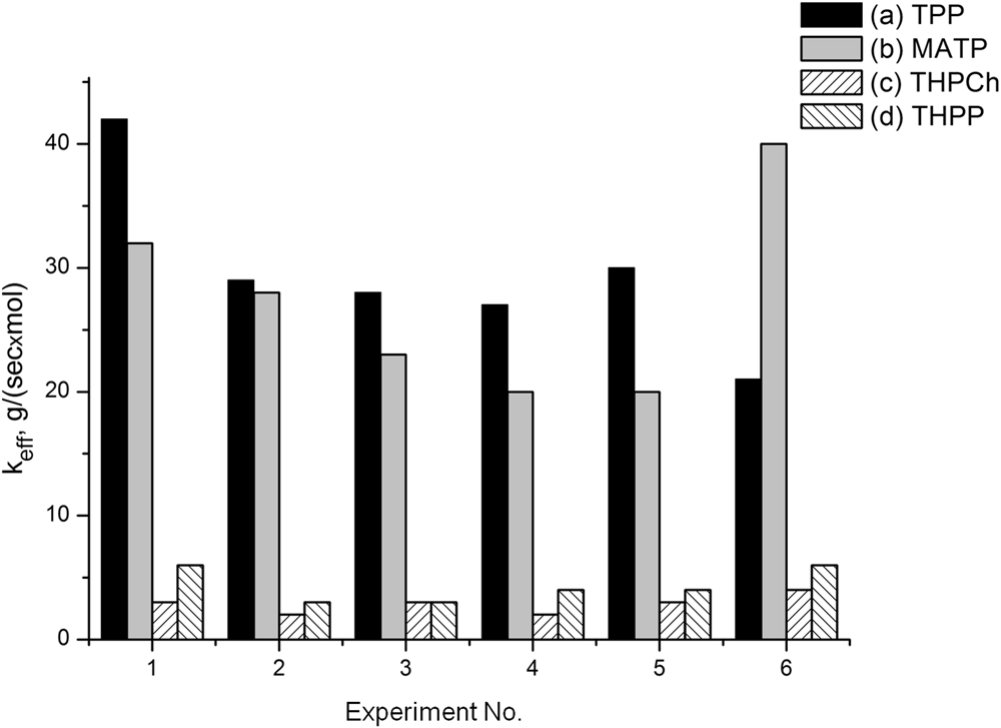
Effective rate constants of tryptophan oxidation for aerogel-porphyrin systems. (**a**) – TPP, (**b**) – MATP, (**c**) – THPCh, (**d**) – THPP. The rate constants were measured in several experiments, repeated in 1 day (6 experiments for TPP and MATP, 2 experiments for THPCh and 3 experiments for THPP). Immobilization of porphyrins on AEG was performed from a chloroform solution.

It is of interest to compare the above effective rate constants *k*_*eff*_ of tryptophan oxidation for the considered systems in respect to the specific surface area of each of the carriers. The specific surface areas of the prepared solid gels measured by the BET method significantly differ. Indeed, the aerogel specific surface area *J*_*ag*_ is 360 m^2^/g, for cryogel and xerogel it is *J*_*cg*_ = 10 m^2^/g and *J*_*xg*_ $\approx$ 1 m^2^/g, correspondingly, which is related to different supramolecular structural organization of the solid gels. We will define the ratios of η = *k*_*eff*_/*J* as specific photocatalytic activities η, expressed in the units of $\chi$ = g^2^(TPP)/[sec × mol(TPP) × m^2^(carrier)]. For the considered systems AEG/TPP, cryogel/TPP and xerogel/TPP we obtain, respectively: η = 0.11, 2.1 and 8.5$\chi$. Thus, the specific photocatalytic activity η for the AEG/TPP system appears to be the lowest one. A higher concentration of the photocatalytic centers – adsorbed TPP molecules or their associates – per surface unit, apparently, corresponds to higher η values. Therefore, with the growth of η, the rate of TPP molecules aggregation reducing the functional activity of the photocatalyst may increase, as well. Moreover, since each catalytic act affects the photocatalytic system’s degradation due to unavoidable dissipation of energy, one may expect increase in the rate of the catalyst degradation with the η parameter growth. It is the factor of the η low value for the PPS immobilized on aerogel (due to the large specific area of AEG) together with the high observed catalytic activity that must a priori determine the high functional stability for the considered AEG/TPP photocatalytic system. Indeed, in our experiments we observed increased stability of the photocatalytic properties for the AEG/TPP system (Fig. 1).

In Fig. 1, we display the effective rate constants *k*_*eff*_ of tryptophan photooxidation in the presence of tetraphenylporphyrins with different meso-substituents immobilized on AEG, obtained in several experiments (see Experimental). These results demonstrate that the systems aerogel/porphyrins retain their initial catalytic activity even during repeated application, i.e. they exhibit significant photostability, in contrast to non-immobilized analogues^20^ and photocatalytic systems with the other two solid gels.

As follows from Fig. 1, TPP appeared to be the most efficient from all the AEG-immobilized porphyrins. At the same time, the comparative activity of the porphyrins immobilized on AEG (TPP ~ MATP > THPCh ~ THPP) coincides with the data on the activity of tetraphenylporphyrins solubilized with pluronics in the homogeneous tryptophan photooxidation in water: TPP ~ MATP > THPP^21^.

To establish the influence of the techniques of porphyrin immobilization on aerogel on the photocatalytic activity of the prepared systems, we measured the rate constants of tryptophan oxidation in the presence of AEG-TPP systems prepared by different techniques (see Experimental). The most efficient photocatalytic systems appeared to be those formed by TPP immobilization on AEG from a chloroform solution. Indeed, for TPP-mediated tryptophan oxidation *k*_*eff*_ = 40.2 g/sec·mol for the mentioned TPP immobilization from a chloroform solution; *k*_*eff*_ = 7.5 g/sec × mol for sc-impregnation; *k*_*eff*_ = 0.5 g/sec × mol for TPP impregnation from isopropanol at the stage of sc-drying of alcogel.

**Figure 2:**
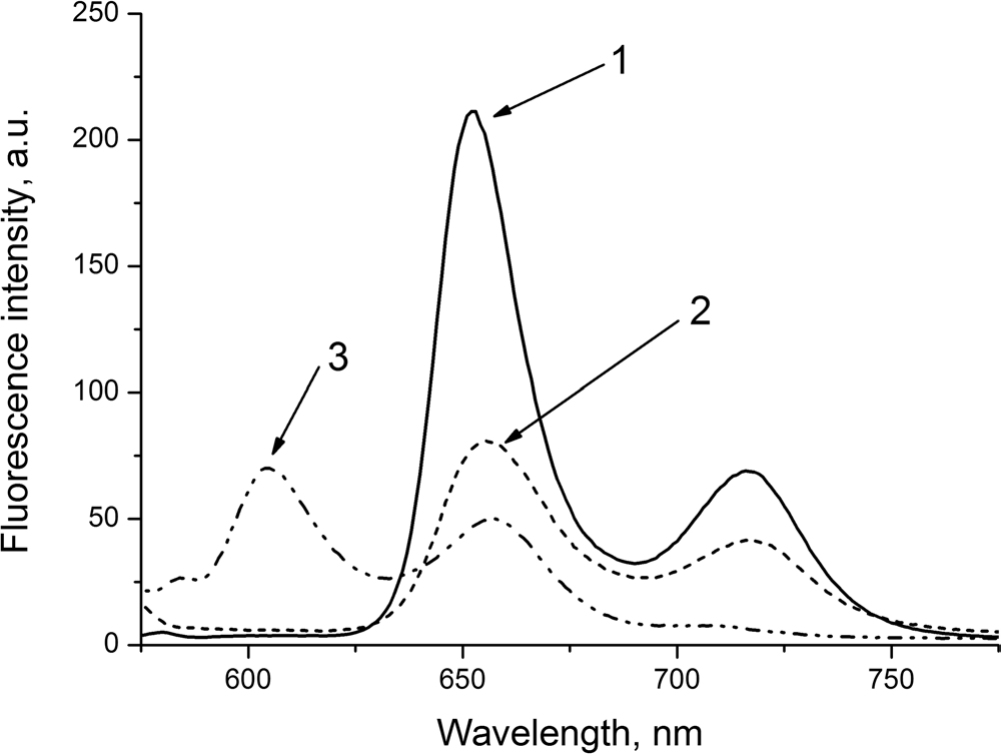
Fluorescence spectra of TPP: 1 – in chloroform solution, 2 – immobilized on aerogel from chloroform, 3 – immobilized on aerogel in scCO_2_.

The drop in the *k*_*eff*_ values during TPP immobilization at the stage of sc-drying of alcogel, or during TPP sc-impregnation in the formed calcium alginate aerogel is apparently caused by the porphyrin interaction with the polysaccharide matrix occurring in both cases, which is accompanied by formation of strong TPP-AEG bonds and porphyrin aggregation. This is testified by the results of the study on the fluorescent properties of the AEG-TPP systems for different techniques of porphyrin immobilization (Fig. 2).

A hypsochromic shift (~50 nm) of the bands in the TPP fluorescence spectrum when TPP is immobilized on AEG in the scCO_2_ medium (3), as compared to the band positions in the initial spectrum of TPP in chloroform (1) and in the spectrum of the porphyrin immobilized on AEG from a chloroform solution (2) indicates aggregation of TPP molecules during sc-impregnation of aerogel, known to result in the non-radiative pathways of the TPP molecules’ excited state relaxation^22–24^.

### The structure of porphyrin-solid gel systems by small-angle X-ray scattering

To determine the structural features of the calcium alginate-based solid gels, we performed an X-ray diffraction study of sodium alginate and solid cross-linked gels on the calcium alginate basis.

**Figure 3:**
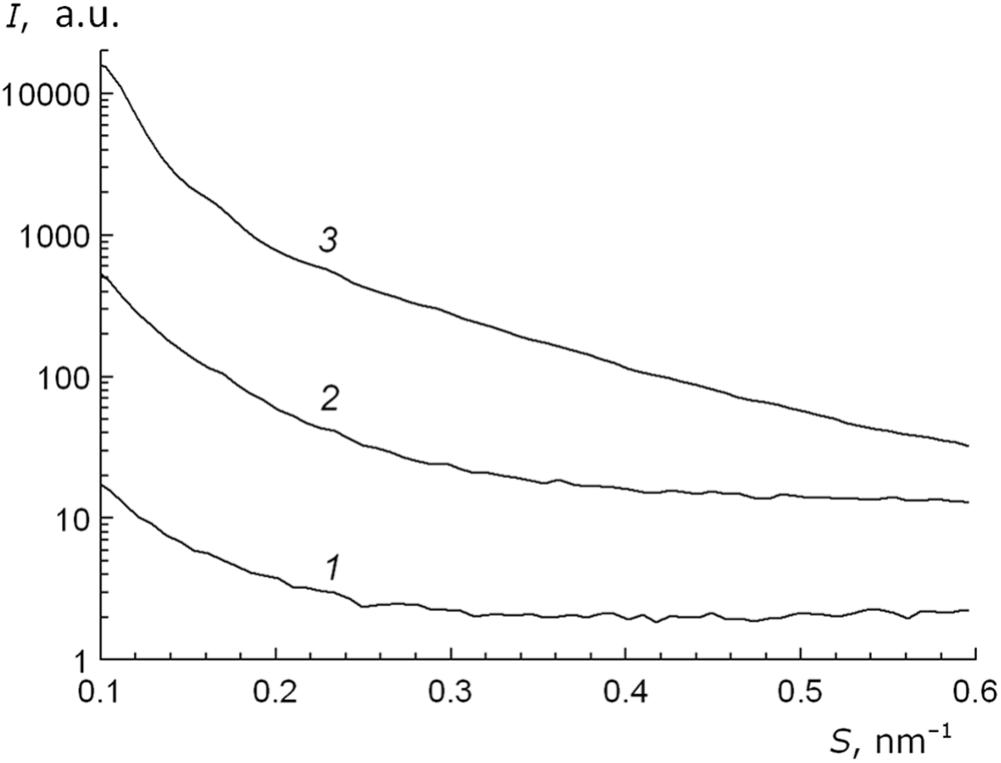
Intensity of small-angle X-ray scattering for a sodium alginate powder (1), calcium alginate xerogel (2) and calcium alginate aerogel (3). A logarithmic scale is used for intensity.

In Fig. 3, we present small-angle X-ray scattering curves for the initial sodium alginate powder, calcium alginate xerogel and aerogel granules, reflecting the information on the structure of those objects at the scale from 2 to 6–7 nm. Sodium alginate has the most homogeneous structure at the mentioned scale (Curve 1), with the lowest X-ray scattering intensity detected. For xerogel samples, the small-angle scattering intensity (Curve 2) exceeds that of sodium alginate by approximately 20 times. This finding testifies formation of inhomogeneities in the xerogel structure at the mentioned scale during isopropanol removal in the conditions of heat drying, when the cross-linked structure of xerogel is formed. Such structural inhomogeneities may account for the higher scattering of X-ray quanta as compared to that of sodium alginate samples. The intensity of small-angle X-ray scattering on aerogel samples increases by one more order of magnitude (Curve 3) in comparison with the scattering on xerogel samples. Such a growth in the scattering intensity is obviously related to the porosity increase in the series of sodium alginate <xerogel <aerogel.

### Thermooxidative destruction studies of solid gels

**Figure 4:**
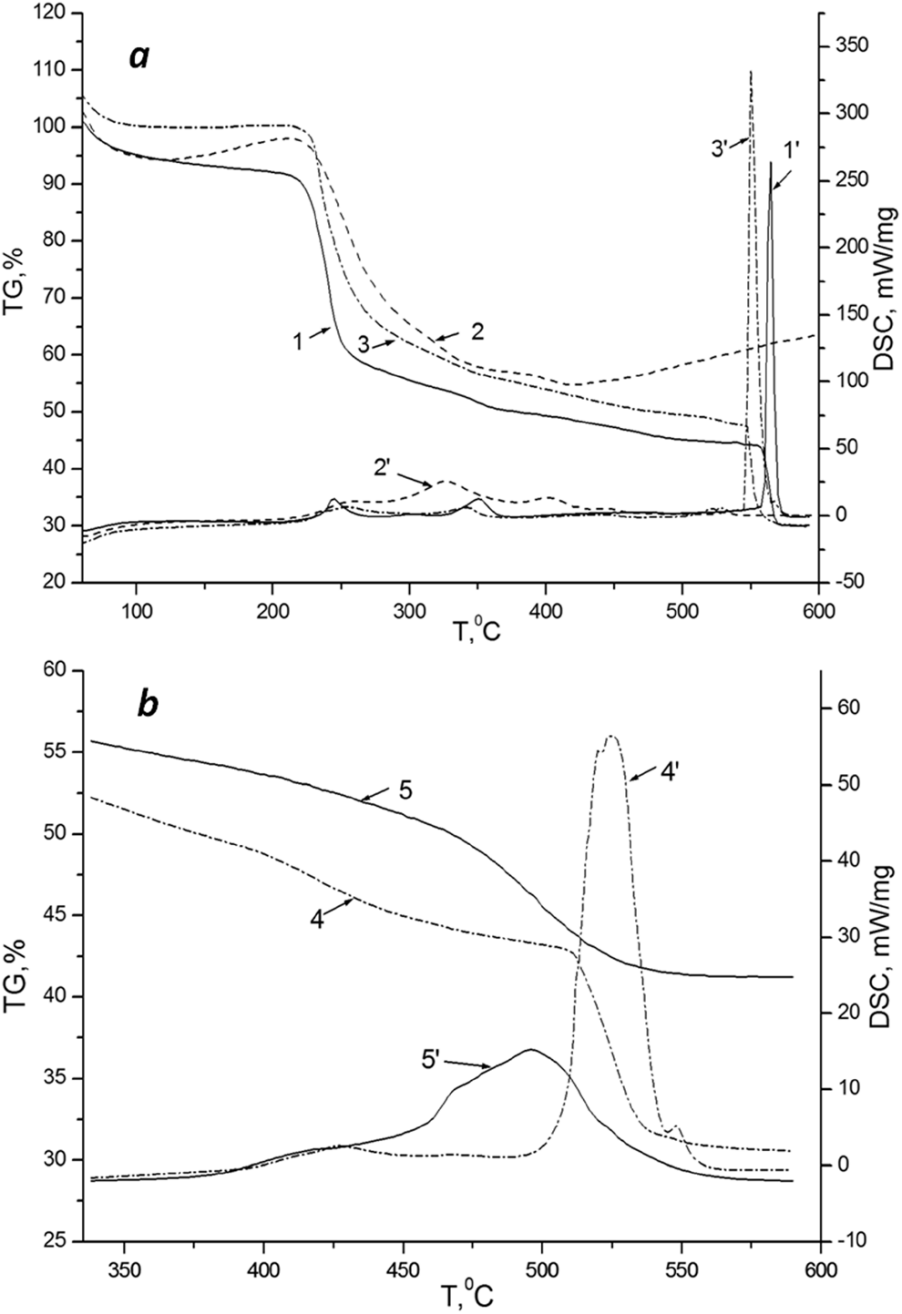
Weight loss plots (TGA) and thermal effects (DSC) of the sodium alginate and solid gels’ TOD in different temperature intervals: (***a***) - initial sodium alginate (1, 1′), calcium alginate aerogel (2, 2′), HA (3, 3′); (***b***) - xero- (4, 4′) and cryogel (5, 5′).

To obtain additional information on the structure of solid gels based on calcium alginate, we have studied thermooxidative destruction (TOD) of calcium alginate aero-, xero- and cryogels, initial sodium alginate and also hyaluronic acid (HA), as a polysaccharide with a structure close to sodium alginate. As seen from Fig. 4а, which depicts the weight loss plots (Curves 1, 2 and 3) and DSC plots (Curves 1′, 2′ и 3′) for the TOD of the initial sodium alginate, calcium alginate aerogel and HA, the main weight loss of these substances due to release of gas components occurs from 200 to 300 °С. The weight loss of xero- and cryogel proceeds in a similar way to sodium alginate. However, the temperature range over 400 °С appeared to be the most interesting for our study. Indeed, in the temperature range of 550–590 °С we detected a high heat flux from 250 to 350 mW/mg for sodium alginate (Curve 1′) and HA (Curve 3′), respectively. The magnitudes of the thermal effects for these processes are presented in Table 1. In the same range, a dramatic weight loss of these samples occurs and formation of the final TOD product – coke residue. At the same time, for the aerogel TOD (Curve 2) we detected a principally different dependence, namely, a monotonous increase of the heat flux from 125 to 250 mW/mg in the temperature range of 400 to 590  °С, as well as some weight gain (of the forming coke residue).

**Table 1:** TOD parameters for sodium alginate, alginate-based cross-linked gels prepared by different techniques of cross-linking and HA in the range of 400–600 °С.

Sample	Δ*m*, %	*Q*, J/g	% coke at 590 °С
Na alginate	20	3180	30
Aerogel	—	40	60
HA	21	5035	30
Cryogel	12	1967	41
Xerogel	18	2847	31

Here Δ*m*,% is a weight loss in the range of 400–600 °С, *Q*, J/g is a thermal effect in the same temperature range, % coke at 590° С is the wt. percent of the coke residue at the temperature of 590 °С.

The plots for the weight loss and heat effects of xerogel (4, 4′) and cryogel (5, 5′) TOD in the temperature range of 350–590 °С are depicted in Fig. 4b. For cryogel the elevated magnitudes of heat flux are found in the temperature range of 450 до 550 °С (the maximum of 15 mW/mg is reached near 500 °С), for xerogel the corresponding interval is shifted to the higher temperature region of 500 to 550 °С (the maximum of 56 mW/mg is reached near 525 °С). Table 1 shows the numeric values of the corresponding thermal effects, as well as the values of other TOD parameters measured for all the studied substances. Besides the weight loss Δ*m* of a sample when heating in the temperature range of 400–600 °С, we also measured the weight of the coke residue after the TOD completion at the temperature of 590 °С. The greatest amount of the coke residue is formed in the aerogel TOD– 60% from the initial weight. For the rest of the studied samples, this value was at the level of 30–40%. At the same time, aerogel, as follows from the data presented in Table 1, was characterized by abnormally low thermal flux (~40 J/g), by almost two orders of magnitude lower than that for cryogel (~2800 J/g) and xerogel (~2000 J/g). The totality of the presented data, along with the data on the unique photostability of porphyrin-impregnated AEG (Fig. 1), which is significantly higher than that for cryogel-TPP and xerogel-TPP systems, unequivocally indicates formation of additional bonds which stabilize the supramolecular organization of aerogel.

## Discussion

The observed characteristics of the immobilized porphyrins’ photocatalytic activity are obviously related to the TPP adsorption activity towards calcium alginate gels, as well as to the details of such systems’ structure. The most important finding is the fact that porphyrins immobilized on calcium-alginate aerogels almost do not lose their activity during multiple (5–7 times) use, while porphyrins dissolved in organic or aqueous media lose their activity rather rapidly^20^. Such a photostability may be associated with the hydrophobicity of the AEG surface which, during photosensitized tryptophan oxidation, restricts the PPS interaction with reactive oxygen species (in particular, water-soluble hydroperoxides) formed as a result of activation of molecular oxygen by excited ^3^PPS* molecules. It is the interaction of such peroxides with porphyrin macrocycles that is believed to be the basic cause of PPS oxidative photodestruction^20^.

The relative activity of heterogeneous (immobilized on AEG) PPS coinciding with the activity of homogeneous (initial) PPS in the anthracene photooxidation, apparently, testifies the absence of participation of tetraphenylporphyrins’ peripheral substituents in their adsorption on AEG and, hence, the binding of porphyrins to the elements of the aerogel surface structure directly via the “plane of four nitrogen atoms” of the macrocycle^25^.

The photocatalytic activity of heterogeneous TPP-solid gel systems is mainly determined by the spatial and surface structure of calcium alginate gels which, in turn, mainly depends on the drying technique, i.e. removal of isopropyl alcohol. During thermal drying, the supramolecular structure of the gel undergoes significant shrinking due to formation of the gas-liquid boundary, which partially destroys intermolecular bonds of the polymer. During freeze drying, the solvent (liquid) freezes inside the pores, and the solvent transition from the solid to the vapor state occurs in the process of drying, without the liquid phase, and essentially lower gel shrinking takes place than that during thermal drying. Solid gels dried in vacuum or on air retain the structure porosity, however, their specific weight is markedly higher, while the specific volume of the inner pores is significantly lower, than those for aerogel^5,26^. Since supercritical fluids combine the properties of liquid and gas, no destruction of the 3D structure of the prepared aerogel by the capillary forces (which appear during the direct removal of the solvent in vacuum or when heating on air) occurs during sc-drying. Thus, aerogels have an almost undeformed supramolecular structure of the initial aqueous gels with the highest porosity and surface area, which apparently facilitates distribution of adsorbed porphyrins in the form of individual molecules or small aggregates with a high catalytic activity. As mentioned before, the specific surface area of aerogel *J*_*ag*_ = 360 m^2^g, while for cryogel *J*_*cg*_ = 10 m^2^/g and for xerogel *J*_*xg*_ $\approx$ 1 m^2^/g.

Such differences are probably associated with a non-uniform solvent removal during drying, while in sc-drying a uniform and gradual complete removal of solvent is achieved, leading to formation of new supramolecular bonds, which strengthen the AEG structure. The growth of small-angle X-ray scattering intensity from xerogel to cryogel to aerogel also indicates formation of most pronounced inhomogeneities in the aerogel structure at the nanometer scale (as compared to xero- and cryogel). The latter may be related to formation of a developed porous structure in such systems in the conditions of drying in the supercritical carbon dioxide medium in an autoclave. One may expect that the enhanced aerogel porosity should result in intensified disaggregation of PPS molecules during their adsorption on the carrier surface and provide the access for the substrate’s molecules to the active centers of the heterogeneous system.

Note that the DTA data testify that the mechanism of thermooxidative destruction changes from xero- and cryogel to aerogel (Fig. 4, Table 1). The alteration of the TOD mechanism may be related to desolvation of the polymer chains’ fragments in the spatially cross-linked calcium alginate during displacement of the liquid phase (isopropyl alcohol) from the gel bulk with the supercritical carbon dioxide. During this process, additional intermolecular bonds may apparently be formed which stabilize the saccharide cycles in the primary structure of the polysaccharide macromolecules. Indeed, the effects of heat release in the temperature range of 500–600 °С, which are obviously associated with the destruction of saccharide cycles in the polysaccharide macromolecules, are rather notable in the cases of sodium alginate and hyaluronic acid, however, they decrease by an almost half of magnitude for xero- and cryogel and are almost negligible in the case of AEG, with the simultaneous increase of the carbonized aerogel (coke residue) fraction (Table 1).

One may expect that the increased porosity of aerogel should facilitate more pronounced disaggregation of the PPS molecules during their adsorption on the carrier surface and provide an access to the active centers of the heterogeneous system for the substrate molecules.

Thus, in this study, we have prepared efficient photocatalytic systems by immobilization of tetraphenylporphyrins on calcium alginate aerogels, with the purpose of creating photocatalysts for substrates’ oxidation in the aqueous phase. We have shown that photocatalytic systems prepared with application of xero- and cryogels as carriers are less active than TPP-AEG systems by more than an order of magnitude.

We believe that such a difference in the photocatalytic activity is related to different spatial and surface structures of the solid gels prepared by different drying techniques of aqueous or alcohol-based gels. Apparently, supercritical drying destroys the spatial structure of hydro- or alcogels to the lowest degree, as compared to cryo- and xerogels prepared in the conditions of thermal drying on air and freeze drying in vacuum. A highly porous AEG structure with the large surface area obviously provides the absence of porphyrin molecules’ aggregation during their immobilization on aerogel leading to enhanced catalytic activity, photostability and a possibility of repeated use of such a catalyst in oxidation processes.

An unexpected result of this study is the establishment of a distinct mechanism of thermooxidative destruction of AEG, as compared to those of initial sodium alginate, xero- and cryogels, as well as of hyaluronic acid (which is similar to sodium alginate). For the mentioned solid gels based on sodium alginate, excluding AEG, a peak of heat emission at 500–600 °С appeared characteristic, while AEG obviously underwent carbonization in the interval of 400–600 °С. It gives rise to a suggestion that additional intermolecular bonds are probably formed in the process of sc-drying, which stabilize glycoside bonds in the polysaccharide molecules, likely via participation of calcium ions. Thus, the TOD character appears an important test providing information for the establishment of aerogel structure of solid polysaccharide gels.

## Methods

### Reagents

**Figure 5:**
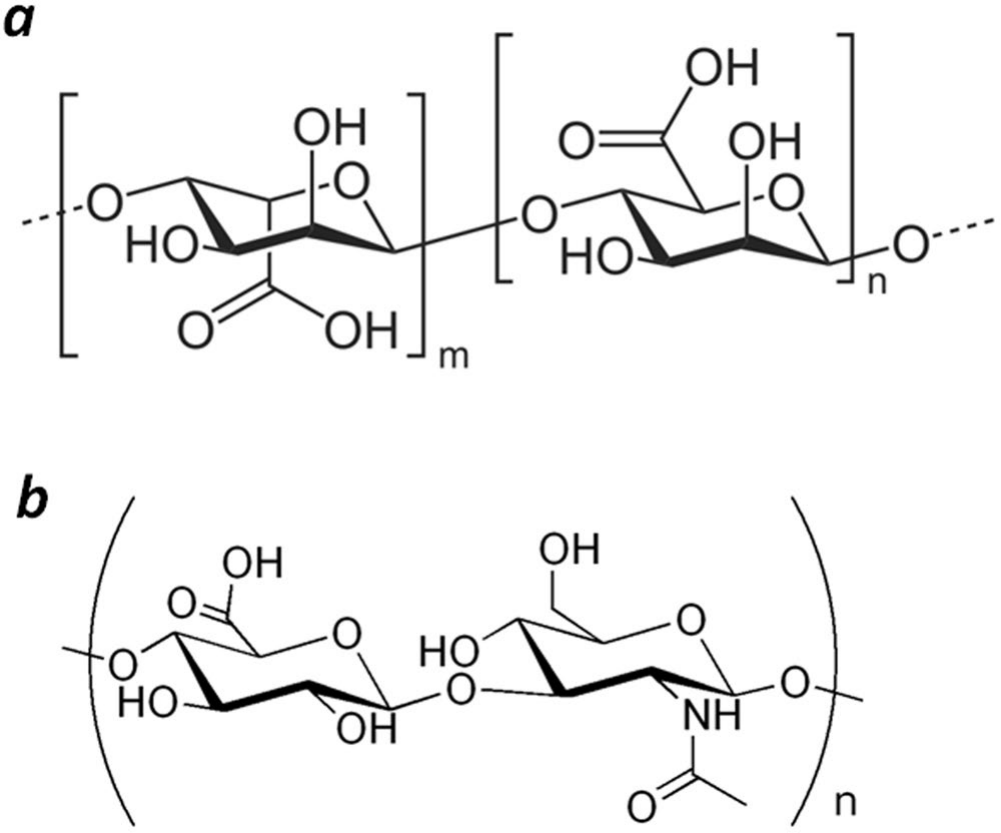
Structural formulae of alginic acid (**a**) and hyaluronic acid (**b**).

All the types of solid gels were prepared based on sodium alginate (Sigma). Calcium chloride (chemically pure) was used as a cross-linker. Isopropanol (chemically pure) was used to prepare alcogels. In the study of thermooxidative destruction of solid gels, we used an amide bond-containing polysaccharide, hyaluronic acid (HA, Sigma), as a reference substance. The structural formulae of these polysaccharides are presented in Fig. 5(a,b).

As porphyrin photosensitizers (PPS), we used a free base tetraphenylporphyrin (TPP, Sigma) and its derivatives – monoaminophenyl,triphenylporphyrin (MATP), tetrahydroxyphenylchlorin (THPCh), tetrahydroxyphenylporphyrin (THPP) synthesized at the Moscow Technological University, Laboratory of Dr. N.A.Bragina.

### Preparation of calcium alginate hydrogel

Calcium alginate solid gels were synthesized from sodium alginate through intermediate synthesis of the corresponding hydro- and alcogels and the following application of different drying techniques^27^. At the first stage, we formed a 3D cross-linked structure of a hydrophobic calcium alginate hydrogel, which existed in a solution in the form of millimeter-sized particles. In doing so, we added a 1% aqueous solution of sodium alginate to a 5% aqueous solution of calcium chloride dropwise under active stirring. The prepared hydrogel stayed in the calcium chloride solution for a day, then it was filtered and washed 7–8 times with an isopropanol-water mixture with a gradual increase of the alcohol content in the mixture from 10–100%.

### Preparation of xero-, cryo- and aerogels

The prepared calcium alginate hydrogel was then subjected to drying. There exist three ways of hydro- and alcogels’ transformation into solid nanostructured highly porous materials: convective (heat) drying, sublimation (freeze) drying and a supercritical treatment. The first two techniques are known to result in deformation of the 3D supramolecular structure of a gel^28^. Thus prepared materials are respectively referred to as xero-, cryo- and aerogels^29,30^. We used all the types of drying, including sublimation (freeze) drying in vacuum for preparation of cryogels and heat drying on air at 50^о^С for 6 hrs for preparation of xerogels. Aerogels were prepared by supercritical drying in the medium of supercritical (sc) carbon dioxide in an autoclave with the volume of 250 cm^3^ at 40 °С and the pressure of 140–145 atm for 8 hrs. As follows from the literature sources^31^ and from our study as well, the use of scCO_2_ allows avoiding destruction of the 3D structure of the prepared aerogel matrix by the surface tension in the process of isopropyl alcohol removal.

### Immobilization of tetraphenylporphyrins on aero-, xero- and cryogels

Immobilization of tetraphenylporphyrins on aerogel was conducted using the following three techniques: dissolution of a porphyrin in isopropyl alcohol, so that, during the subsequent alcohol substitution with scCO_2_, the porphyrin was adsorbed on the aerogel surface (“sc-drying”); impregnation of AEG with the PPS molecules in the scCO_2_ medium (“sc-impregnation”); deposition from a PPS solution in chloroform. In the first case, we used a high pressure reactor with the volume of 60 cm^3^ at 50^о^С and a pressure of 120 atm. The weight of the porphyrin sample in the reactor was 5 mg, the aerogel weight was 30 mg, the treatment duration was 1 hr. Then the reactor was cooled to room temperature, and carbon dioxide was removed for 10 min. The prepared AEG with adsorbed PPS was washed with chloroform to remove free PPS. The content of PPS introduced in AEG in the scCO_2_ medium was (0.5–1) × 10^−5^ mol/g.

To adsorb PPS from a chloroform solution, AEG (10 mg) was kept in a porphyrin solution in chloroform for 1 day followed by washing the prepared heterogeneous photocatalyst with chloroform to remove free PPS.

**Figure 6:**
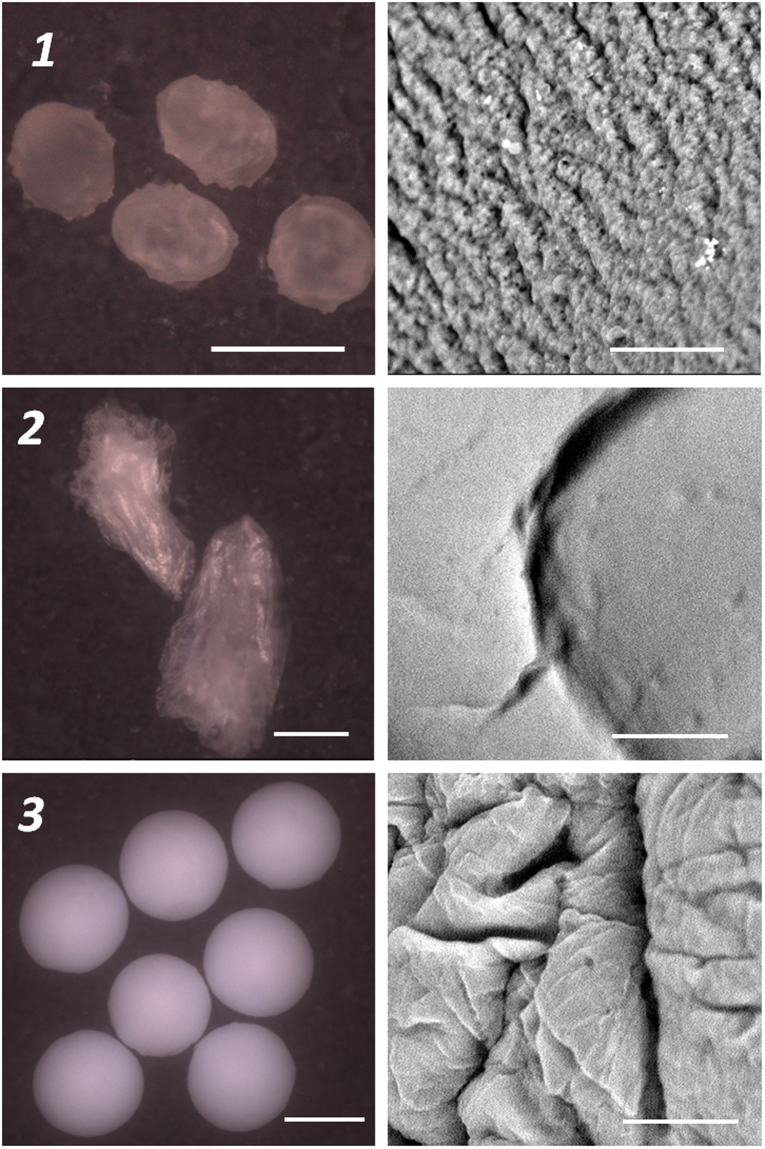
SEM micrographs of the prepared solid gels: (1) – xerogel, (2) – cryogel, (3) – aerogel. *Left panel* - general view of particles (Bar = 1 mm), *Right panel* – details of the surface (Bar = 10 µm).

PPS immobilization on xero- and cryogels was conducted from a PPS solution in chloroform in a similar manner. The prepared photocatalytic systems had a form of spherical beads of a 1–3 mm diameter (Fig. 6).

We measured the PPS content in AEG from UV-Vis absorption spectra, by the difference in the Soret band intensity in the initial porphyrin solution and in the same solution after the completion of immobilization, also taking into account the amount of porphyrin released into the solution during washing of the aerogel particles containing adsorbed PPS. The porphyrins content in AEG was (0.5–3.0) × 10^−6^ mol/g.

### Photocatalytic activity measurements

We measured the photocatalytic activity of the prepared systems using oxidation of tryptophan as a test substrate in water, adding particles of aerogels with immobilized porphyrins to a tryptophan aqueous solution (1 × 10^−4^ М) in a cuvette (3 mL). The weight of AEG, cryo- and xerogel particles was 10 mg. The content of immobilized porphyrins in solid gels was (1–30) × 10^−7^ mol/g. The solution with AEG was stirred until no changes in the absorption spectrum of tryptophan were observed (for about 30 min) for the establishment of the equilibrium in the system (in the beginning of stirring, tryptophan was partially adsorbed on aerogel, so its concentration in water was somewhat diminished). After the tryptophan concentration in the reaction mixture had stabilized, we switched the illumination on and started the count of the reaction time. The illumination of the reaction mixture was performed with an AFS light-diode apparatus (Polironik Ltd, Russia), with the working wavelength of λ = 400 nm and the power of 210 mWt. Similarly, we conducted the reaction of tryptophan photooxidation with PPS immobilized on xero- and cryogel. The PPS content in cryo- and xerogel varied within the same range as that for aerogel.

The observed rate constant *k*_*obs*_ of tryptophan photooxidation was determined by the relative change in the substrate concentration in the solution in the initial time interval Δ*t* (sec) of the process. The effective rate constant *k*_*eff*_ was defined as the ratio of *k*_*obs*_ to the photosensitizer concentration in the aerogel particles (mol/g), thus *k*_*eff*_ = Δ*C*_*s*_ /(*C*_*s*_ *C*_*P*_Δ*t*), where *C*_*P*_ was the concentration of PPS in the aerogel particles in mol/g, *C*_*s*_ and Δ*C*_*s*_ were the tryptophan concentrations at the zero time and its decrease for the time Δ*t*, respectively.

After the reaction completion, the AEG beads with immobilized porphyrins were removed from the reaction system, rinsed with water, dried and used repeatedly in tryptopan oxidation in the similarly prepared systems containing tryptophan. The time between such *k*_*eff*_ measurements was 1 day. We conducted 6 measurements in total.

### Differential thermal analysis (DTA)

The differential thermal analysis (DTA) of the samples was performed with the use of a STA 449 F3 simultaneous thermal analyzer by the NETTZCH. The destruction process was conducted on air at the gas flow rate of 30 mL/min and the linear heating rate of 10 °С/min, the samples’ charges were 5–10 mg. The weight loss was registered with the accuracy up to 10^−3^ mg, the relative error of the temperature measurement was ± 1.5 °С. We acquired the plots of the weight loss (TGA) and the thermal effects (DSC) on the temperature. The values of the coke residue (coke, wt.%) and the heat flux in the different temperature ranges (*Q*, J/g) were used as the quantitative characteristics of the thermooxidative destruction of polysaccharides and solid gels.

### Small-angle X-ray scattering

The information on the structure of the formed systems was obtained based on the analysis of small-angle X-ray scattering intensity measured using a diffractometer with a position-sensitive detector^32,33^. The experimental intensity of scattering for each specimen was corrected taking into account the background scattering, normalized to the specimen weight and represented as a function of *I*(*S*), where *S* = (2sin*θ*)/*λ*, 2*θ* is the scattering angle, *λ* is the X-ray radiation wavelength (for the used CuKα radiation *λ* = 1.542 Å).

### Specific surface area measurements

We measured the specific surface area of the solid gels using low-temperature (80 К) argon adsorption. A closed quartz vessel with the specimen inside was filled with a given amount of the gas. After the establishment of the equilibrium pressure, decreased (as compared to the initial pressure) as a result of argon adsorption on the specimen’s surface, we detected the number of adsorbed gas molecules (Δ*N*) and the equilibrium pressure (*Р*) of argon. Then, another portion of argon was added to the vessel. The result of the measurement was a plot of Δ*N* = Δ*N*(*Р*), i.e. the plot of the number of adsorbed argon molecules vs. its equilibrium pressure. The experimental data analysis was performed within the framework of the BET equation.

### Spectroscopy

UV-Vis absorption spectra of samples were recorded with a Cary 50 (Varian) spectrophotometer. Fluorescence spectra and fluorescence excitation spectra of samples were recorded using a Cary Eclipse (Varian) spectrofluorometer.

### Data availability

No datasets were generated or analysed during the current study.
